# Acylcarnitine enrichment as a characteristic of rheumatoid arthritis fibroblast-like synoviocyte metabolic fingerprint

**DOI:** 10.1016/j.jtauto.2025.100310

**Published:** 2025-08-19

**Authors:** Georgios K. Vasileiadis, Yuan Zhang, Marion Laudette, Tahzeeb Fatima, Anna-Karin Hultgård Ekwall, Reshmi Sureshkumar, Ronald van Vollenhoven, Jon Lampa, Bjorn Gudbjornsson, Espen A. Haavardsholm, Dan Nordström, Gerdur Gröndal, Kim Hørslev-Petersen, Kristina Lend, Merete L. Hetland, Michael Nurmohamed, Mikkel Østergaard, Till Uhlig, Tuulikki Sokka-Isler, Anna Rudin, Jan Borén, Monica Guma, Cristina Maglio

**Affiliations:** a Department of Rheumatology and Inflammation Research, Institute of Medicine, Sahlgrenska Academy, University of Gothenburg, Gothenburg, Sweden; b Department of Molecular and Clinical Medicine/Wallenberg Laboratory, Institute of Medicine, Sahlgrenska Academy, University of Gothenburg, Gothenburg, Sweden; c Department of Rheumatology, Sahlgrenska University Hospital, Gothenburg, Sweden; d Department of Medicine, Rheumatology Unit, Center for Molecular Medicine (CMM), Karolinska Institute, Karolinska University Hospital, Stockholm, Sweden; e Amsterdam Rheumatology and Immunology Center, Amsterdam University Medical Center, Amsterdam, Netherlands; f Centre for Rheumatology Research, Landspitali University Hospital, Reykjavik, Iceland; g Faculty of Medicine, University of Iceland, Reykjavik, Iceland; h Center for treatment of Rheumatic and Musculoskeletal Diseases (REMEDY), Diakonhjemmet Hospital, Oslo, Norway; i Faculty of Medicine, University of Oslo, Oslo, Norway; j Division of Medicine and Rheumatology, Helsinki University Hospital, Helsinki, Finland; k University of Helsinki, Helsinki, Finland; l Danish Hospital for Rheumatic Diseases, University Hospital of Southern Denmark, Sønderborg, Denmark; m Department of Regional Health Research, University of Southern Denmark, Odense, Denmark; n Copenhagen Center for Arthritis Research (COPECARE) and DANBIO, Center for Rheumatology and Spine Diseases, Rigshospitalet, Glostrup, Denmark; o Department of Clinical Medicine, Faculty of Health Sciences, University of Copenhagen, Copenhagen, Denmark; p Amsterdam Rheumatology and Immunology Center, Reade, Amsterdam, Netherlands; q University of Eastern Finland, Kuopio, Finland; r Hospital Nova, Wellbeing Services County of Central Finland, Jyväskylä, Finland; s Sahlgrenska University Hospital, Gothenburg, Sweden; t Department of Medicine, University of California San Diego, La Jolla, CA, USA

**Keywords:** Rheumatoid arthritis, Fibroblast-like synoviocytes, Metabolomics, Acylcarnitines

## Abstract

**Objective:**

In rheumatoid arthritis (RA), fibroblast-like synoviocytes (FLS) alter their metabolism to support their activation. We aimed to analyse the full spectrum of metabolic alterations associated with RA by performing untargeted metabolomics in RA FLS vs. non-inflamed (NI) FLS.

**Methods:**

Untargeted annotated metabolomics was performed using mass spectrometry on ten primary RA and seven NI FLS culture extracts and 220 serum samples from participants with early RA from the randomised controlled NORD-STAR trial. Carnitine-related proteins were measured with Western blot. FLS bioenergetic profile was assessed with a Seahorse flux analyser.

**Results:**

Metabolomics analysis based on 138 annotated metabolites revealed a distinct metabolic fingerprint between RA and NI FLS. Of the 12 metabolites enriched in RA FLS, 11 were acylcarnitines. Pro-inflammatory stimulation of NI FLS also led to acylcarnitine accumulation. RA FLS exhibited lower levels of CD36, a fatty acid transporter, but similar levels of L-carnitine transporter, and carnitine palmitoyltransferase 1 A and 2 compared to NI FLS. Seahorse analyses showed no difference in fatty acid oxidation between RA and NI FLS; however, RA FLS displayed mitochondrial dysfunction and energetic impairment. Serum acylcarnitine content decreased after 24 weeks of treatment with methotrexate combined with abatacept or tocilizumab in patients with early RA achieving remission.

**Conclusion:**

Acylcarnitine accumulation is a characteristic of RA FLS metabolic fingerprint and could be linked to mitochondrial dysfunction. In patients with early RA, acylcarnitine content in serum decreases after successful anti-rheumatic treatment. These results indicate a dysregulation in acylcarnitine metabolism in RA at the joint level and systemically.

## 1 Introduction

Fibroblast-like synoviocytes (FLS) are mesenchymal cells residing on the synovial membrane of the joints [1]. The physiological role of FLS is to lubricate and nourish surrounding cartilage surfaces by regulating extracellular matrix and synovial fluid homeostasis. They are also essential for connective tissue development and homeostasis under normal and inflammatory conditions [2]. In the inflamed synovium of rheumatoid arthritis (RA), however, the synovial membrane transforms into a hyperplastic pannus-like structure of proliferating FLS and macrophages that extend into the joint space, reaching the cartilage to invade and degrade the cartilage matrix [3].

The trans-differentiation of 10.13039/100010135FLS from anabolic quiescence in health to a chronically activated pathogenic phenotype in RA triggers cellular metabolic reprogramming to provide the energy needed to support such activation [1,4]. For instance, glycolysis is upregulated and supports an aggressive phenotype in RA 10.13039/100010135FLS [3,[5], [6], [7], [8]]. Glutamine and choline metabolism have also been shown to be enhanced in RA FLS compared to osteoarthritis FLS [[9], [10], [11]]. Furthermore, mitochondrial respiration is impaired in RA FLS, making these cells less metabolically flexible [7,12].

The main targets for disease-modifying anti-rheumatic drugs (DMARDs) currently in use are cytokines and immune cells; the development of drugs specifically targeting FLS is still an unmet need in RA [3]. The metabolic pathways associated with FLS activation could be therapeutically targetable, but further studies are required to uncover their potential as drug targets [6,13]. Moreover, it is still unknown whether metabolic alterations in RA FLS are a consequence of the disease or whether they might contribute to early-stage disease pathogenesis [4].

In the current work, we aimed to uncover the full spectrum of metabolic alterations in RA FLS by performing untargeted annotated metabolomics analysis with liquid chromatography coupled to quadrupole time-of-flight mass spectrometry (LC-QTOF-MS) in RA FLS vs. non-inflamed (NI) FLS from individuals with no previous history of arthritis. Our goal was to identify novel metabolites that characterise the RA FLS metabolic fingerprint and expand current knowledge on the metabolic and bioenergetic alterations in RA FLS. To validate our findings, we have also examined serum samples from an independent cohort of people with untreated early RA.

## 2 Methods

### 2.1 Cell cultures

**Fig. 1:**
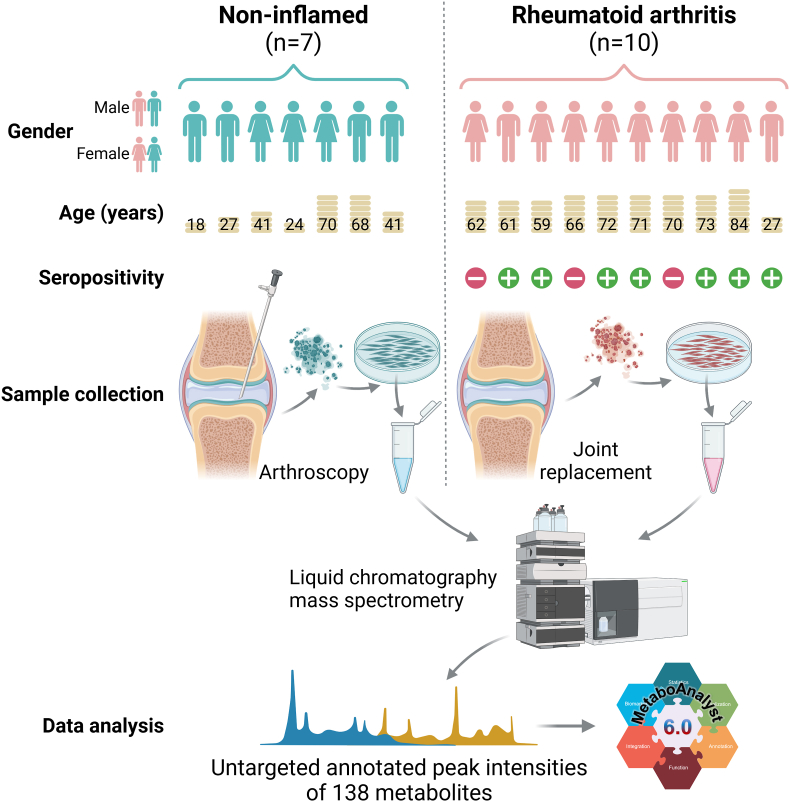
**FLS metabolomics set-up overview.** Created in https://BioRender.com.

The study plan is detailed in Fig. 1. RA FLS were isolated from synovial tissue from patients with RA, undergoing joint replacement surgery at Sahlgrenska University Hospital, Sweden. All RA patients fulfilled the American College of Rheumatology 2010 classification criteria for the disease [14]. NI FLS were isolated from patients with no previous history of arthritis, undergoing diagnostic arthroscopy due to a previous injury that occurred over 2 months prior to the operation; no sign of macroscopic inflammation or swelling was present at the time of the arthroscopy. Details on FLS isolation and culture are shown in Supplementary Methods. All study participants gave their written informed consent, and the studies were approved by the regional ethics board in Gothenburg (1087-16; 573-07; 334-15; 2019–04373, 2020–07116, and 2022-01977).

### 2.2 Metabolomics by LC-QTOF-MS in FLS

FLS (passage 7) were detached using 0.05 % Gibco™ Trypsin-EDTA and 200,000 cells/well were seeded into each well of 6-well plates in 1 ml growth medium and incubated at 37 °C with 5 % CO_2_ for 24h. The medium was then changed to new growth medium without stimulation or with 10 ng/ml tumour necrosis factor (TNF; Sigma, St. Louis, MO, USA) or 10 ng/ml interleukin 1β (IL-1β; Sigma, St. Louis, MO, USA) and incubated at 37 °C for 16h. Cells were washed rapidly in ice-cold PBS and harvested with cell scrapers in 500 μl of 80 % methanol (HPLC-grade Methanol, Fisher Scientific) per well and 400 μl of the cell suspension from each well was transferred to pre-chilled 1.5 ml micro tubes on ice and stored at −80 °C until their transportation to the Swedish Metabolomics Centre in Umeå, Sweden for metabolic profiling by LC-QTOF-MS. Details on chromatographic separation, and mass spectrometry are shown in Supplementary Methods.

### 2.3 Metabolomics by LC-QTOF-MS in the NORD-STAR cohort

Nordic Rheumatic Diseases Strategy Trials and Registries (NORD-STAR) is a multicentre, randomised, longitudinal trial, comparing active conventional anti-rheumatic treatment vs. three different biological DMARDs in untreated early RA. The complete details of the study are provided elsewhere [15,16]. Briefly, study participants were randomized (1:1:1:1) into four treatments at baseline. All participants received methotrexate (MTX) up to 25 mg/week, and they also received oral prednisolone in arm 1; certolizumab-pegol in arm 2; abatacept in arm 3; and tocilizumab in arm 4. The primary endpoint was response to treatment at 24 weeks defined by Clinical Disease Activity Index remission (CDAI≤2.8) [15,16]. All study participants gave their written informed consent, and the study was approved by the regional ethics board in Stockholm (2011/2069-31/4; amendment: 2020–03797).

For the purpose of this study, a subset of 220 participants (58, 52, 56, and 54 for arms 1, 2, 3, and 4, respectively) was randomly selected from the Swedish arm (n = 393) of the NORD-STAR cohort to perform the metabolomic profiling [17]. Their serum samples were aliquoted (100 μl) and stored at −80 °C until their transportation to the Swedish Metabolomics Centre for metabolic profiling by LC-QTOF-MS. Details on chromatographic separation, and mass spectrometry are shown in Supplementary Methods.

### 2.4 Western blot

FLS (passage 6–9) were detached using 0.05 % Gibco™ Trypsin-EDTA, seeded into each well of 6-well plates in 1 ml growth medium (100,000 cells/well), and incubated overnight. Details on cell lysate collection, protein electrophoresis, transferring, and used antibodies are shown in Supplementary Methods. Images were acquired using Odyssey XF Imager (LI-COR, Lincoln, NE, USA) and the qualitative analysis of the target proteins was performed using Empiria Studio 3.0 software (LI-COR). Whole cell lysate of skin fibroblasts was used as an internal control to compensate for the results obtained from different membranes.

### 2.5 Seahorse assays

We used a Seahorse flux analyser to measure the mitochondrial oxygen consumption rate (OCR) and the extracellular acidification rate (ECAR) in real-time with live FLS (passages 6–9). ATP Rate Assay, Fatty Acid Oxidation, and Mito Stress tests were used according to the manufacturer's instructions (Agilent Technologies, Santa Clara, CA, USA). For all assays, 6–8 (3–4 for Fatty Acid Oxidation) replicates of FLS were seeded in a XF96-well microplate at a density of 15,000 cells/well in high glucose (4.5 g/L) growth medium and incubated at 37 °C with 5 % CO_2_ for 16 h. After 16 h the medium was replaced with high or low glucose (1 g/L) growth medium to induce glucose deprivation. After 24 h, seahorse experiments were performed (Supplementary Methods).

### 2.6 Statistical analysis

Metabolomics analysis was performed with MetaboAnalyst 6.0 and GraphPad Prism version 10 (GraphPad Software, San Diego, CA, USA). The peak intensities of metabolites detected in FLS were normalised with autoscaling. The peak intensities of metabolites detected in NORD-STAR serum samples were first standardised using internal control metabolites for both positive and negative modes and then normalised with autoscaling. Regarding metabolite clustering, Euclidean distance was used to measure distance, and Ward's linkage as a clustering algorithm. The discrimination in acylcarnitine content between RA and NI FLS was evaluated with the area under the receiver operating characteristic curve (ROC-AUC). Differences in metabolite content were analysed with Mann-Whitney test, 2-way ANOVA, mixed-effects analysis, or Wilcoxon test, when appropriate. Seahorse analyses were performed with Seahorse Wave software (Agilent Seahorse Bioscience, RRID: SCR_014526). Parameters were calculated following the instructions of Seahorse XF Report Generator for each respective test and unpaired *t*-test was used to compare two groups. Paired *t*-test and unpaired *t*-test were used in NORD-STAR serum samples. A p < 0.05 was considered statistically significant.

## 3 Results

### 3.1 Acylcarnitine enrichment is a characteristic of RA FLS metabolic fingerprint

**Fig. 2:**
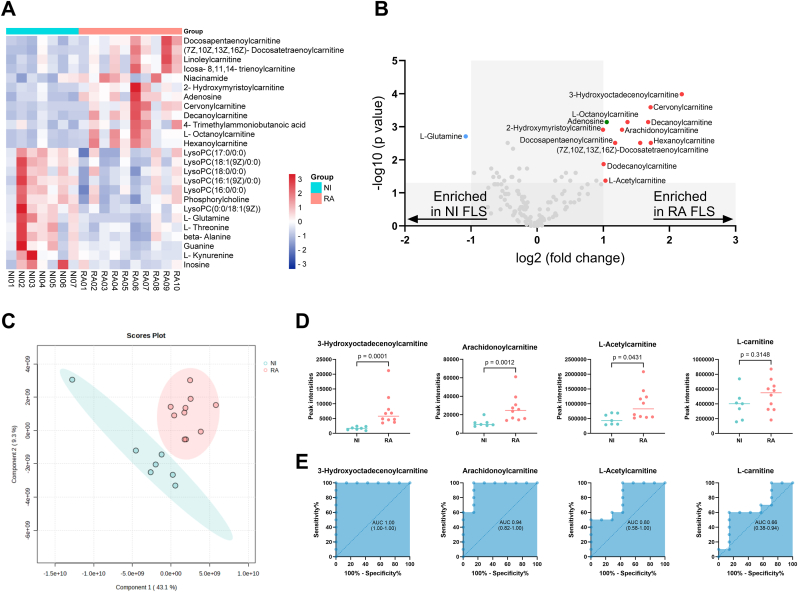
**FLS metabolomics analysis. A**. Clustering result shown as a heatmap. Distance measure using Euclidean distance. Samples are shown on the x axis, while metabolites are shown on the y axis. **B.** Enriched metabolites by volcano plot defined as fold change (x) ≥2 and Mann-Whitney test p-value (y) < 0.05. The red dots represent acylcarnitines. **C**. Partial least squares - discriminant analysis showing the 2-D scores plot between principal component 1 and 2. Each dot represents a sample. The cycles show the 95 % confidence interval. **D**. Dot plots of the peak intensities of three selected acylcarnitines and L-carnitine in NI and RA FLS. Mann-Whitney test was performed to determine the significance. **E**. Receiver operating characteristic curves of three selected acylcarnitines and L-carnitine. Sensitivity is on the y-axis, and the specificity on the x-axis. Results are from 10 RA FLS and 7 NI FLS. Abbreviations: RA, rheumatoid arthritis; NI, non-inflamed; FLS, fibroblast-like synoviocytes; AUC, area-under-the-curve.

The characteristics of the study participants donating FLS are detailed in Fig. 1 and Supplementary Table 1. Peak intensities of 138 annotated metabolites were acquired from 10 unstimulated RA FLS and 7 unstimulated NI FLS (Fig. 1). Metabolite peak intensities were normalised with auto scaling (Supplementary Fig. 1A) and principal component analysis of principal components 1 and 3 is shown in Supplementary Fig. 1B. Metabolomics analysis identified a distinct metabolic fingerprint between unstimulated RA and NI FLS as illustrated by hierarchical clustering shown as a heatmap, and partial least squares–discriminant analysis (Fig. 2A–C). A volcano plot combining fold change analysis (≥2) and Mann-Whitney test p values (<0.05) revealed 13 metabolites having different levels in unstimulated RA and NI FLS; 12 were enriched in RA FLS, while one was enriched in NI FLS (Fig. 2B). Of the 12 metabolites enriched in RA FLS, 11 were acylcarnitines, fatty acid–carnitine conjugates, and the other was adenosine (Fig. 2B). We also identified glutamine as reduced in RA FLS. The specific acylcarnitines and their classifications are shown in Supplementary Table 1, where acylcarnitines of all lengths are represented. Fig. 2D shows univariate analysis and ROC curves of three selected acylcarnitines and L-carnitine between unstimulated RA and NI FLS. Taken together, a significant accumulation of acylcarnitines, but not L-carnitine, was observed in RA FLS compared to NI FLS.

### 3.2 Pro-inflammatory stimulation induces acylcarnitine enrichment in NI FLS

**Fig. 3:**
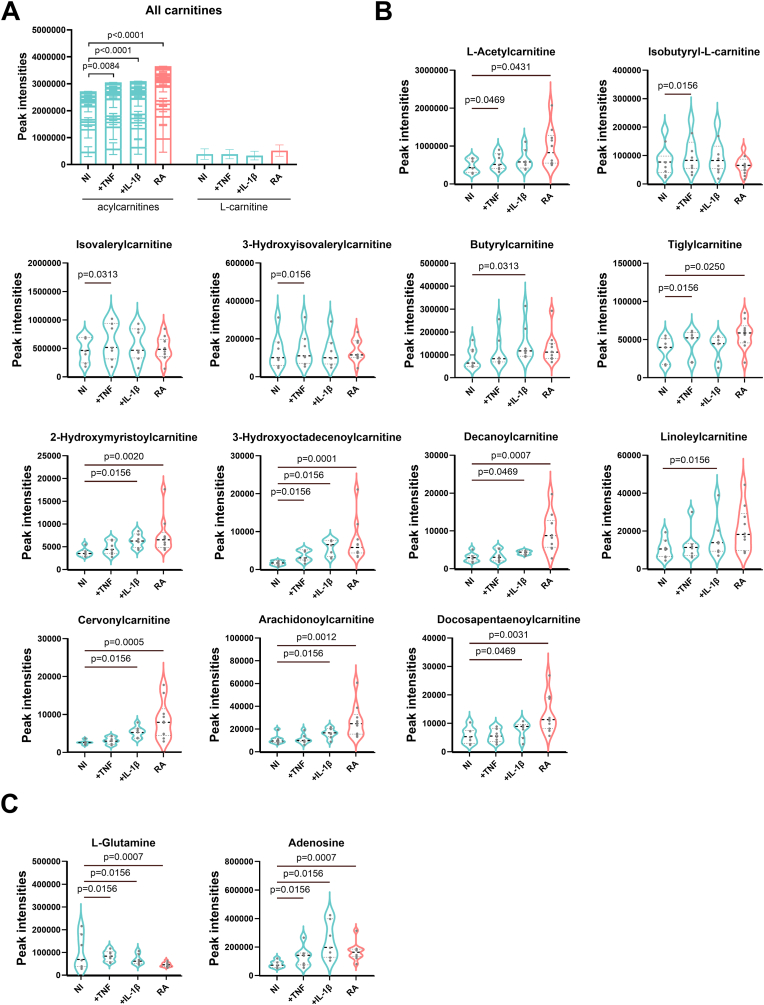
**Acylcarnitine species, L-carnitine, L-glutamine, and adenosine in FLS**. **A.** Average peak intensities of all identified acylcarnitine species stacked together and L-carnitine, **B.** significantly changed acylcarnitines, **C.** L-glutamine, and adenosine in NI-FLS unstimulated or stimulated with 10 ng/ml TNF or 10 ng/ml IL-1β for 16h prior to sample collection, as well as in RA FLS. Differences between groups were calculated using autoscaled data with 2-way ANOVA in **A** and Wilcoxon test in **B** and **C** to compare unstimulated and stimulated NI FLS, or Mixed effects model in **A** and Mann-Whitney test in **B** and **C** to compare between NI and RA FLS. Results are from 10 RA FLS and 7 NI FLS. Abbreviations: RA, rheumatoid arthritis; NI, non-inflamed; FLS, fibroblast-like synoviocytes; TNF, tumour necrosis factor; IL-1β, interleukin 1β.

Having uncovered that RA FLS accumulated acylcarnitines intracellularly, we next wanted to determine whether acylcarnitine accumulation was inducible in NI FLS by pro-inflammatory stimuli. We stimulated NI FLS with TNF or IL-1β for 16h followed by metabolomics analysis, as above. Both stimuli induced an increase in total acylcarnitine content but not L-carnitine in NI FLS compared to unstimulated NI FLS (Fig. 3A). Levels of thirteen acylcarnitines were significantly increased after FLS activation (Fig. 3B). Changes of L-glutamine and adenosine were shown in Fig. 3C. These results indicate that acylcarnitine accumulation is not only present in chronically inflamed RA FLS but is also inducible, to a certain extent, in NI FLS by various pro-inflammatory stimuli.

### 3.3 Fatty acid influx is altered in RA FLS, but L-carnitine transporter and enzymes regulating acylcarnitine formation are unchanged

**Fig. 4:**
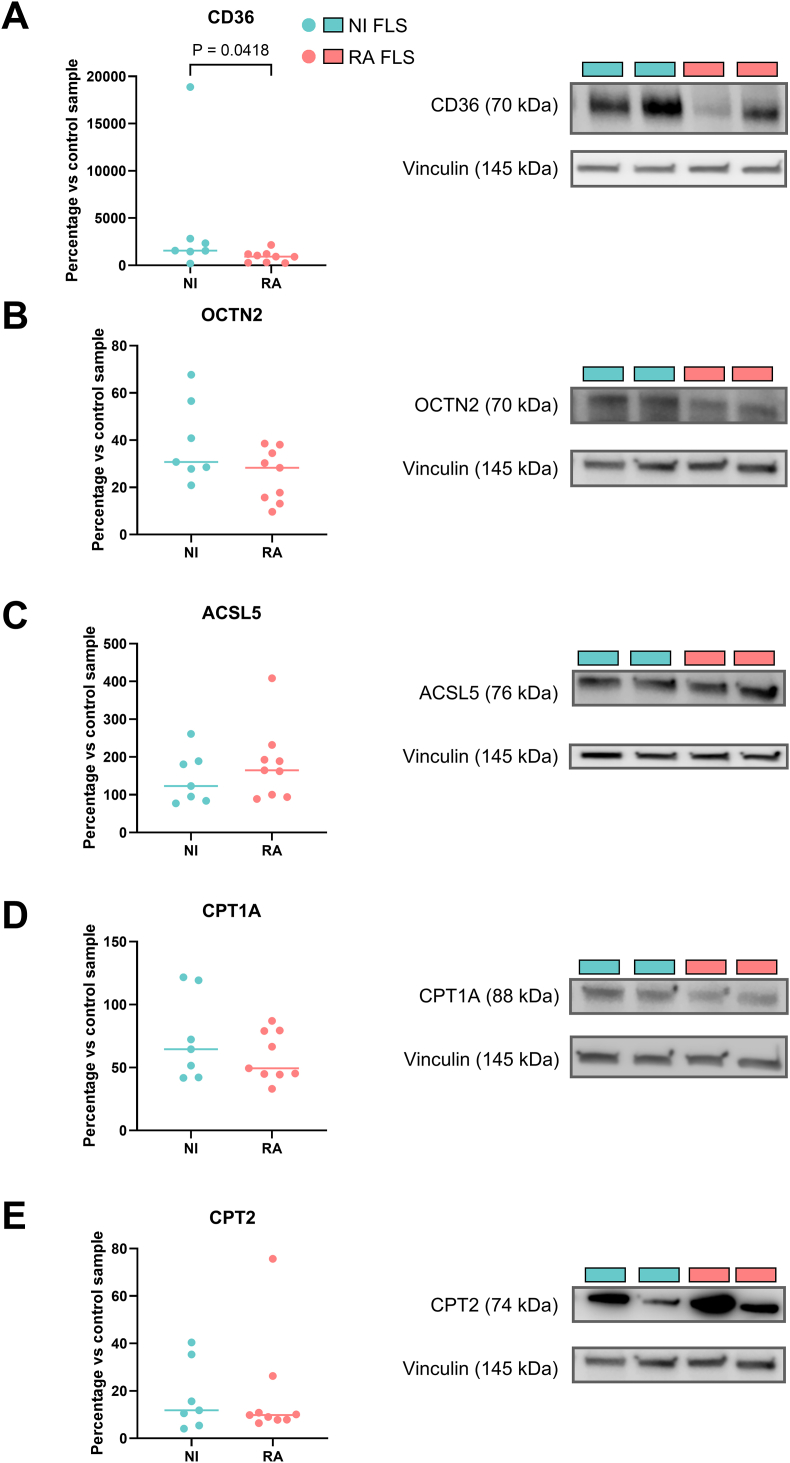
**Expression of acylcarnitine-related transporters and enzymes.** Expression of **A.** CD36 (cell membrane transporter of fatty acids), **B.** OCTN2 (cell membrane transporter of L-carnitine), **C.** ACSL5, **D.** CPT1A, and **E.** CPT2 shown as bar graphs and representative images. Data were normalised to vinculin and expression levels are shown as percentages of the same control sample loaded to different gels. Significance was determined with Mann-Whitney test. Results are from 9 RA FLS and 7 NI FLS. Abbreviations: OCTN2, solute carrier family 22 member 5; ACSL5, long-chain-fatty-acid—CoA ligase 5; CPT, carnitine palmitoyl transferase.

We next wanted to establish if acylcarnitine accumulation is the result of alterations in the expression of acylcarnitine-related transporters and enzymes. We firstly measured the levels of CD36 and OCTN2, cell membrane transporters of fatty acids and L-carnitine respectively, to determine if acylcarnitine accumulation was the result of increased uptake of L-carnitine and/or fatty acids by the cells. Expression of CD36, but not OCTN2, significantly decreased in RA FLS compared to NI FLS (Fig. 4A and B, Supplementary Fig. 2). Next, we measured ACSL5, CPT1A, and CPT2. ACSL5 is an isozyme that activates free long-chain fatty acids into acyl-coenzyme A (CoA) esters. The acyl groups can then be transferred to L-carnitine in a reaction catalysed by CPT1A to form acylcarnitines. After transportation of the acylcarnitines into the mitochondria, acylcarnitines will be broken by CPT2 into a fatty acid, which undergoes fatty acid oxidation (FAO) for energy production, and L-carnitine (that is exported from the mitochondrion). No significant difference in ACSL5, CPT1A, or CPT2 expression was found in RA vs. NI FLS (Fig. 4C–E, Supplementary Fig. 2). Having found a decreased level of CD36 in RA FLS compared to NI FLS with similar expression levels of OCTN2, ACSL5, CPT1 and CPT2, we conclude that the enrichment of acylcarnitines in RA FLS is not caused by elevated levels of acylcarnitine-related transporters or enzymes.

### 3.4 Acylcarnitine accumulation is not linked to higher energy production from fatty acids in RA FLS

**Fig. 5:**
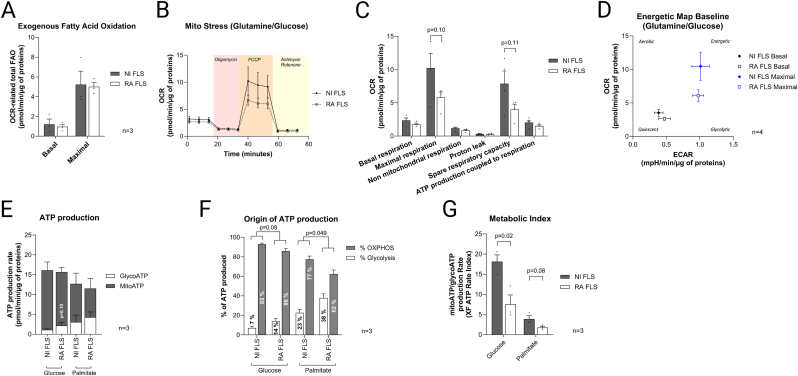
**Seahorse analysis. A**. Quantification of total FAO at baseline and under FCCP-induced stress (maximal) with the Fatty Acid Oxidation test. **B**. OCR kinetics, **C**. quantification of respiratory parameters, and **D**. energetic maps obtained by plotting ECAR and OCR values at baseline and under FCCP-induced stress of RA vs NI FLS using glucose/glutamine as substrate with the Mito Stress test. **E**. GlucoATP (ATP production associated with conversion of glucose to lactate) and MitoATP (ATP production associated with OXPHOS in the mitochondria) production rates (pmol ATP/min/μg of proteins), **F**. percentages of total ATP and **G**. metabolic index calculated as the ratio of mitoATP/glycoATP production rate with the use of ATP Rate Assay test. In all tests, OCR was measured under basal conditions and after addition of oligomycin (1 μM) to inhibit ATP synthase, FCCP (1 μM) to uncouple OXPHOS, and antimycin A (2 μM) and rotenone (2 μM) to calculate non-mitochondrial respiration. Data were obtained from 4 – 8 replicates for each independent sample (3–4 samples per group). Data were normalised to total protein content and shown as mean ± SEM and p values obtained from unpaired *t*-test.

Acylcarnitines’ main function is to transport fatty acids into the mitochondria to be oxidised for energy production through FAO. Our next step was thus to determine whether acylcarnitine accumulation in RA FLS was associated with altered FAO. We used the Fatty Acid Oxidation test with Seahorse flux analyser in real-time with live cells. The test showed that basal and maximal OCR-related FAO were unaltered between RA and NI FLS (Fig. 5A). These results indicate that the increased acylcarnitine content in RA FLS does not translate into a higher energy production via the utilisation of fatty acids by the mitochondria of these cells.

### 3.5 RA FLS display energetic impairment

We used the Mito Stress test with Seahorse to assess the mitochondrial function in RA FLS compared to NI FLS in real-time with live cells. We showed that RA FLS compared to NI FLS presented a lower maximal respiration, and spare respiratory capacity, but due to high inter-individual variation these results were not statistically significant (Fig. 5B and C). RA and NI FLS had unchanged basal respiration, proton leak, and ATP production coupled to respiration when glucose and glutamine were the substrates (Fig. 5B and C). Furthermore, RA FLS had a lower oxidative bioenergetic profile (OCR) under maximal respiration conditions compared to NI FLS, indicating energetic impairment (Fig. 5D).

Finally, by simultaneously quantifying glycolytic and mitochondrial ATP production rates with the Seahorse ATP real-time rate assay, we assessed the glycolysis and oxidative phosphorylation (OXPHOS) rates in RA FLS and NI FLS (Fig. 5E). The total ATP production rates and proportions of ATP generated by glycolysis and OXPHOS in a glucose-enriched environment were similar between the two groups (Fig. 5E and F). However, proportions of ATP generated in RA FLS shifted more from OXPHOS to glycolysis under a low glucose environment as compared to NI FLS (Fig. 5F). Interestingly, under a low glucose environment, when palmitate was used as substrate, both NI and RA FLS had higher proportions of ATP generated by glycolysis compared to when glucose was used as substrate, suggesting again a dependency on glucose compared to fatty acids for this cell type (Fig. 5F). Importantly, the metabolic index (indicating whether the metabolic phenotype is more oxidative or more glycolytic) was significantly reduced in RA FLS vs. NI FLS when cultured with glucose – and showed a similar trend when cultured with palmitate – indicating a less oxidative and more glycolytic phenotype in RA FLS (Fig. 5G).

Taken together, the results indicate that RA FLS might not use the higher acylcarnitine content for energy production, but rather display energetic impairment caused by mitochondrial dysfunction. This metabolic impairment could be responsible for the accumulation of acylcarnitines in these cells.

### 3.6 RA and NI FLS exhibit similar responses to acylcarnitine overload

To determine if the enriched acylcarnitines in RA FLS could provide energy to the cells, we stimulated both NI and RA FLS with three exogenous acylcarnitines including acetylcarnitine (short chain), decanoylcarnitine (medium chain), and stearoylcarnitine (long chain). Among those three exogenous acylcarnitines, acetylcarnitine and decanoylcarnitine were found enriched in RA FLS, and their levels increased in TNF or IL-1β stimulated NI FLS (Fig. 2, Fig. 3B), and stearoylcarnitine tended to be enriched in RA FLS (Mann-Whitney p = 0.11, fold change 1.56 compared to NI FLS) and to be upregulated by IL-1β stimulation in NI FLS (Wilcoxon test p = 0.08). We observed no difference between NI and RA FLS stimulated with different concentrations of all three exogenous acylcarnitines in the cell viability determined by Cell Counting Kit 8 assay and IL-6 production measured using ELISA and, indicating that NI and RA FLS react similarly to an overload of acylcarnitines (Supplementary Fig. 3).

### 3.7 Circulating levels of acylcarnitines in NORD-STAR participants with early RA are reduced after treatment

**Fig. 6:**
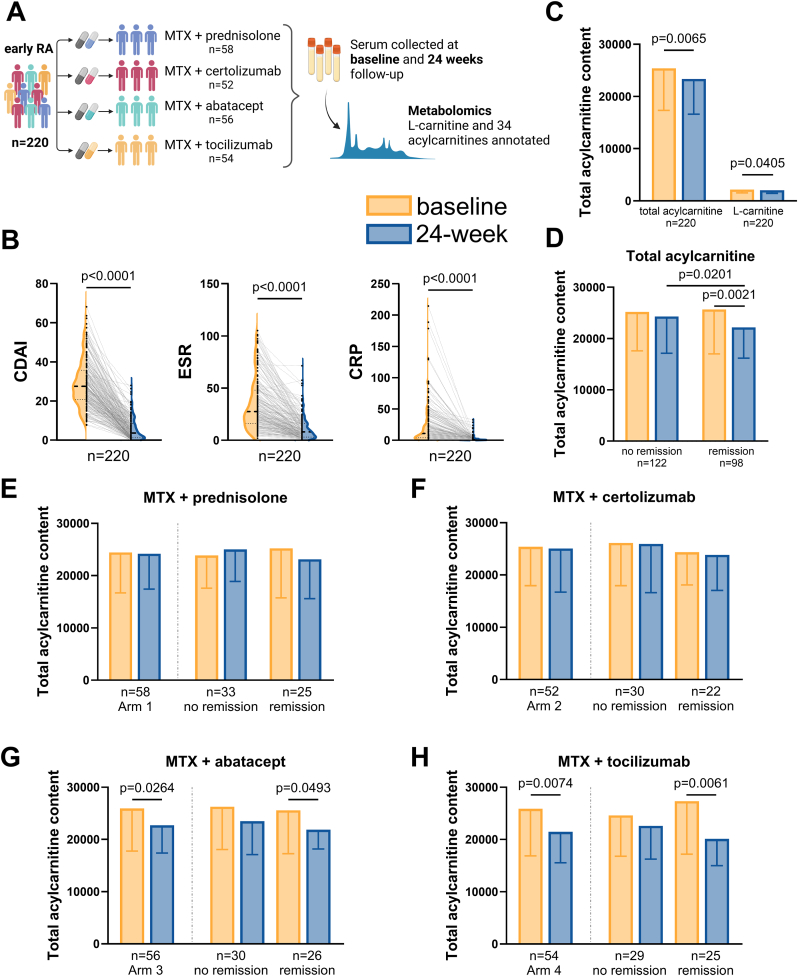
**Circulating acylcarnitines detected by metabolomics in participants with early RA in NORD-STAR study. A.** Study description and metabolomics overview. Created in https://BioRender.com. **B.** Changes of inflammatory markers ESR, CRP, and CDAI before and after treatment. **C.** Total acylcarnitine content defined as the sum of all the detected acylcarnitines' peak intensities and L-carnitine at baseline and 24-week follow-up. **D.** Total acylcarnitine content after stratifying for remission at 24 weeks (CDAI≤2.8). **E-H.** Total acylcarnitine content at baseline and 24-week follow-up in each treatment arm and after stratifying for remission. Significance was determined with paired *t*-test for comparisons before and after treatment or unpaired *t*-test for comparisons between remission and non-remission. Abbreviations: MTX, methotrexate; ESR, erythrocyte sedimentation rate; CRP, C-reactive protein; CDAI, Clinical Disease Activity Index.

To determine if acylcarnitine metabolism is systemically affected in RA, we analysed the metabolomics data of L-carnitine and 34 detected acylcarnitines obtained from serum samples of 220 NORD-STAR participants (Fig. 6A and Supplementary Table 2). At baseline, total acylcarnitine content, defined as the sum of all the detected acylcarnitines’ peak intensities, was positively associated with erythrocyte sedimentation rate (ESR) but not C-reactive protein (CRP), CDAI, or swollen joint count (SJC) (Supplementary Fig. 4). Along with markers of inflammation, such as CDAI, ESR, and CRP (Fig. 6A), total acylcarnitine content decreased after 24 weeks of treatment (Fig. 6B and Supplementary Fig. 5 with CDAI in treatment arms). When stratifying for CDAI remission at 24 weeks, total acylcarnitine content significantly decreased after treatment only in participants who achieved remission (CDAI≤2.8, n = 98, Fig. 6C). Among all four treatment arms, we observed a reduction of total acylcarnitine content after treatment in arm 3 (MTX + abatacept), and 4 (MTX + tocilizumab) but not in arm 1 (MTX + prednisolone) and 2 (MTX + certolizumab-pegol) (Fig. 6E–H). When stratifying for remission at 24 weeks, reduction of total acylcarnitine content was also only observed in participants who achieved remission in arm 3 and 4 (Fig. 6E–H). Taken together, these results suggest that circulating acylcarnitine content in serum is reduced following successful anti-rheumatic treatment with MTX + abatacept or MTX + tocilizumab.

## 4 Discussion

This study aimed to identify the metabolic alterations defining the metabolic fingerprint of RA FLS. By performing MS-based metabolomics in RA FLS vs. NI FLS from people with no previous history of arthritis, we identified a distinct metabolic profile in RA FLS, characterised by an enrichment in acylcarnitines. This accumulation was inducible in NI FLS after pro-inflammatory stimulation. Despite elevated acylcarnitine levels, RA FLS did not exhibit an increase in mitochondrial FAO but showed energetic impairment linked to mitochondrial dysfunction. Finally, MS-based metabolomics in patients with early RA showed that acylcarnitine content was reduced following successful DMARD treatment.

Metabolomics in cells can help understand the molecular mechanisms behind disease pathophysiology by providing an accurate and highly dynamic screenshot of the cellular phenotype [18]. In fact, cells alter their metabolism under pathogenic conditions to adapt to the environmental and their new metabolic needs [19]. Several metabolic pathways, such as glycolysis, glutaminolysis, and choline metabolism, have been described as upregulated in RA FLS vs. FLS collected from people with osteoarthritis [5,[7], [8], [9], [10], [11]]. However, osteoarthritis FLS are not an ideal control for RA FLS, as they show signs of inflammation [20]. Our control FLS were isolated from people without any history of arthritis, at least two months following an injury, and in the absence of any macroscopic inflammation or swelling. For these reasons, NI FLS serve as a better control against RA FLS. Only one previous metabolomics study analysed cell lysates from RA vs. NI FLS [21] using Nuclear Magnetic Resonance spectroscopy and identifying 36 metabolites, of which only glycerol was found reduced in RA FLS. By contrast, our metabolomics analysis with LC-QTOF-MS identified 138 metabolites, 13 of which had different levels between RA and NI FLS. The set-up of that study was different compared to ours as the authors included RA FLS from resolving arthritis together with NI FLS and early RA FLS. This, together with the different methods used to perform metabolomics, might account for the different number of metabolites detected between the two studies.

Acylcarnitines are formed by combining one molecule of fatty acid with one molecule of L-carnitine by CPT1 and they are de-conjugated by CPT2. Their main purpose is to transport long-chain fatty acids across the mitochondrial membranes to be oxidised for energy production [22]. Acylcarnitines can also be formed in the peroxisomes, to export products of the peroxisomal β oxidation, which usually results in long-chain acylcarnitines that then enter the mitochondria to continue the FAO [22]. Here, we demonstrated that RA FLS, and NI FLS after pro-inflammatory stimuli accumulate acylcarnitine intracellularly compared to unstimulated NI FLS. To further investigate the causes of this accumulation, we measured the transporters and enzymes related to acylcarnitine anabolism. Whereas the L-carnitine transporters OCTN2 levels were similar between RA and NI FLS, the fatty acid transporters CD36 decreased RA FLS, suggesting a negative feedback mechanism. Moreover, our results showed that acylcarnitine accumulation in RA FLS was not dependent upon CPT1 or CPT2. In addition, in the Seahorse experiments assessing FAO, we used etomoxir, a CPT1A inhibitor and observed similar results in RA and NI FLS, further indicating that RA and NI FLS have similar CPT1A activity. Other enzymes, such as carnitine acetyltransferase and carnitine octanoyltransferase, can create acylcarnitines; however, the majority of our acylcarnitines have chain lengths of 10 or more carbons indicating that they were created by CPT1, rather than those enzymes, which are mostly related to acylcarnitines with shorter chain lengths [22].

Our Seahorse experiments in live cells showed that total FAO was unchanged between RA and NI FLS despite the higher acylcarnitine load in the former, suggesting that RA FLS cannot efficiently process the enriched acylcarnitines. Furthermore, RA FLS had energetic impairment caused by mitochondrial dysfunction. Previous studies in different contexts have shown that mitochondrial dysfunction can cause or aggravate acylcarnitine accumulation [23,24]. A potential mechanism for that is the APT production from dysfunctional mitochondria is reduced and that leads to activation of CPT1 [22]. It has been proposed that, as acylcarnitine accumulation does not induce any feedback inhibition of CPT1, acylcarnitine levels continue to increase over time [22]. In such conditions of FAO or OXPHOS defects, acylcarnitines with medium and long chain lengths are the main species that are enriched [23]. Specifically, out of 11 acylcarnitines that were enriched in RA FLS, three were long-chain (including 3-Hydroxyoctadecenoylcarnitine that was the most altered metabolite detected), and four were medium chain acylcarnitines. Of interest, a recent Mendelian randomisation study identified 13 metabolites as causal risk factors for RA, and 11 of which are in the lipid metabolic pathways [25]. Taken together, our results indicate that RA FLS accumulate acylcarnitines that they cannot efficiently process, due to dysfunctional mitochondria.

Mitochondrial dysfunction is a hallmark of RA FLS and plays a role in RA pathogenesis [26]. Dysfunctional mitochondria contribute to the production of pro-inflammatory cytokines leading to increased proliferation in RA FLS [27,28]. Notably, it has been shown that induced mitochondrial dysfunction leads to inflammatory responses in NI FLS and even sensitises these cells to IL-1β stimulation, amplifying the resulting inflammatory response [29]. In our study, RA FLS accumulated acylcarnitines that they could not efficiently process due to mitochondrial dysfunction, a condition that is linked to proinflammatory responses. FLS stimulation with acylcarnitines did not generate distinct responses in RA vs NI FLS, suggesting that the accumulation of acylcarnitines in RA FLS is more likely the result rather than the cause of mitochondrial dysfunction in these cells. In addition to being a result of mitochondrial dysfunction, acylcarnitine accumulation can have important cellular implications *per se*. As an example, long-chain acylcarnitines decrease mitochondrial respiration, pyruvate, and lactate oxidation, as well as induce ROS production and mitochondrial damage [22,30,31]. Importantly, acylcarnitine stimulation, but not L-carnitine or fatty acids, induced activation of nuclear factor kappa B in murine macrophage cell lines [23]. Moreover, long-chain acylcarnitines induced IL-6 production and cell death in murine myoblast cell lines [30]. Our results showed that one of the most abundant groups of acylcarnitines enriched in RA FLS was indeed the long-chain acylcarnitines. One of those is arachidonoylcarnitine, a precursor of arachidonic acid, a long-chain fatty acid involved in inflammation in RA [32,33]. Taken together, acylcarnitine accumulation, either because of mitochondrial dysfunction or intrinsically, could aggravate inflammation in the context of RA FLS, but further studies are needed to confirm this.

To determine if acylcarnitine metabolism is affected systemically in RA, we performed LC-QTOF-MS to identify L-carnitine and acylcarnitines in serum samples of 220 people with early untreated RA from the NORD-STAR study, both at baseline and 24 weeks following four different treatment arms. Previous studies have demonstrated that long-chain acylcarnitine levels are increased in people with RA compared to healthy controls, while short-chain acylcarnitines are inversely associated with the future incidence of RA in women [34,35]. A recent study identified 24 acylcarnitines as risk factors for RA using a machine learning model [36]. Our serum metabolomics analysis suggests that acylcarnitine metabolism is affected not only in RA FLS, but also systemically. The systemic elevation of acylcarnitines in patient serum likely represents integrated contributions from multiple cellular compartments. In patients with early RA, anti-rheumatic treatment reduced acylcarnitine content only in participants achieving remission, while in those who did not achieve remission, it remained unchanged. When stratifying for treatment arm, a decrease in serum acylcarnitine content was observed in participants achieving who received treatment with MTX + abatacept or MTX + tocilizumab rather than those who received MTX + prednisolone or MTX + certolizumab-pegol. However, due to the small sample size in each treatment arm, we cannot exclude that MTX, which was given to all the participants, together with prednisolone or certolizumab-pegol, might also have an impact on total acylcarnitine content. Little is known about the effect of abatacept and tocilizumab on acylcarnitine metabolism. A previous study in RA has shown that abatacept may affect genes involved in mitochondrial function [37]. Tocilizumab, as an IL-6 receptor antagonist, is known to suppress oxidative stress and therefore may improve mitochondrial function [38]. These findings open up future investigations to elucidate the relationship between acylcarnitine metabolism and DMARDs treatment in the context of RA.

Together with an increase in acylcarnitines in RA FLS, we also found adenosine enriched in RA FLS vs. NI FLS. Adenosine is a metabolite produced in response to hypoxia, metabolic stress, or injury [39] and elevated intracellular adenosine has been linked to low cellular energy charge [40]. A previous study has reported high adenosine levels in RA synovial fluid compared to osteoarthritis [41]. We also found glutamine decreased in RA FLS vs NI FLS, which is likely the result of increased glutaminolysis [10].

The major limitation of our study is that we only performed metabolomics in whole cells, not in isolated mitochondria. Consequently, we do not know if the identified acylcarnitines are located inside the mitochondria, or in the cytosol. Nevertheless, it has been suggested that accumulated fatty acid intermediates of FAO are exported from the mitochondria to the cytosol to protect them from toxicity via CPT2 [42].

In summary, by performing LC-QTOF-MS in RA vs NI FLS, we showed that acylcarnitine enrichment is a characteristic of RA FLS metabolic fingerprint. We demonstrated that RA FLS have an energetic impairment and mitochondrial dysfunction, which could be the cause of acylcarnitine accumulation in these cells. In a large patient cohort of early RA, we found that circulating acylcarnitines are reduced after successful anti-rheumatic treatment, at least in participants receiving MTX + abatacept or MTX + tocilizumab.

## CRediT authorship contribution statement

**Georgios K. Vasileiadis:** Writing – review & editing, Writing – original draft, Methodology, Investigation, Formal analysis, Data curation, Conceptualization. **Yuan Zhang:** Writing – review & editing, Writing – original draft, Methodology, Investigation, Formal analysis, Data curation, Conceptualization. **Marion Laudette:** Writing – review & editing, Methodology, Investigation. **Tahzeeb Fatima:** Writing – review & editing, Data curation, Conceptualization. **Anna-Karin Hultgård Ekwall:** Writing – review & editing, Data curation, Conceptualization. **Reshmi Sureshkumar:** Writing – review & editing, Methodology, Investigation. **Ronald van Vollenhoven:** Writing – review & editing, Resources, Data curation. **Jon Lampa:** Writing – review & editing, Resources, Data curation. **Bjorn Gudbjornsson:** Writing – review & editing, Resources, Data curation. **Espen A. Haavardsholm:** Writing – review & editing, Resources, Data curation. **Dan Nordström:** Writing – review & editing, Resources, Data curation. **Gerdur Gröndal:** Writing – review & editing, Resources, Data curation. **Kim Hørslev-Petersen:** Writing – review & editing, Resources, Data curation. **Kristina Lend:** Writing – review & editing, Resources, Data curation. **Merete L. Hetland:** Writing – review & editing, Resources, Data curation. **Michael Nurmohamed:** Writing – review & editing, Resources, Data curation. **Mikkel Østergaard:** Writing – review & editing, Resources, Data curation. **Till Uhlig:** Writing – review & editing, Resources, Data curation. **Tuulikki Sokka-Isler:** Writing – review & editing, Resources, Data curation. **Anna Rudin:** Writing – review & editing, Resources, Data curation. **Jan Borén:** Writing – review & editing, Data curation, Conceptualization. **Monica Guma:** Writing – review & editing, Data curation, Conceptualization. **Cristina Maglio:** Writing – review & editing, Validation, Supervision, Project administration, Funding acquisition, Data curation, Conceptualization.

## Funding statement

This study was supported by the 10.13039/501100004359Swedish Research Council (nr 2021-01442), 10.13039/501100003748Swedish Society for Medical Research (nr S20-0109), the 10.13039/501100004063Knut and Alice Wallenberg Foundation and the 10.13039/501100017018Wallenberg Centre for Molecular and Translational Medicine at the 10.13039/501100005760University of Gothenburg, the Swedish federal government under LUA/ALF agreement concerning research and education of doctors (ALFGBG-965478 and ALFGBG-978776), Konung Gustav 10.13039/100001368V Foundation, and the Swedish Association Against Rheumatism (R-969009 and R-982136) to Cristina Maglio.

## Declaration of competing interest

The authors declare the following financial interests/personal relationships which may be considered as potential competing interests:Anna-Karin Ekwall reports a relationship with AbbVie Inc that includes: board membership and consulting or advisory. Anna-Karin Ekwall reports a relationship with Boehringer Ingelheim Pharma GmbH & Co KG that includes: speaking and lecture fees. Anna-Karin Ekwall reports a relationship with Pfizer Inc that includes: board membership. Ronald van Vollenhoven reports a relationship with Alfasigma SpA that includes: funding grants. Ronald van Vollenhoven reports a relationship with AstraZeneca Pharmaceuticals LP that includes: consulting or advisory, funding grants, and speaking and lecture fees. Ronald van Vollenhoven reports a relationship with BMS that includes: consulting or advisory, funding grants, and speaking and lecture fees. Ronald van Vollenhoven reports a relationship with Galapagos NV that includes: consulting or advisory, funding grants, and speaking and lecture fees. Ronald van Vollenhoven reports a relationship with MSD France SAS that includes: funding grants. Ronald van Vollenhoven reports a relationship with Novartis Pharmaceuticals Corporation that includes: funding grants. Ronald van Vollenhoven reports a relationship with Pfizer Inc that includes: consulting or advisory, funding grants, and speaking and lecture fees. Ronald van Vollenhoven reports a relationship with Roche SAS that includes: funding grants. Ronald van Vollenhoven reports a relationship with Sanofi that includes: funding grants. Ronald van Vollenhoven reports a relationship with ucb that includes: consulting or advisory, funding grants, and speaking and lecture fees. Ronald van Vollenhoven reports a relationship with AbbVie Inc that includes: consulting or advisory and speaking and lecture fees. Ronald van Vollenhoven reports a relationship with biogen that includes: consulting or advisory and speaking and lecture fees. Ronald van Vollenhoven reports a relationship with GSK that includes: consulting or advisory and speaking and lecture fees. Ronald van Vollenhoven reports a relationship with Janssen that includes: consulting or advisory and speaking and lecture fees. Ronald van Vollenhoven reports a relationship with remegen that includes: consulting or advisory and speaking and lecture fees. Bjorn Gudbjornsson reports a relationship with Novartis that includes: consulting or advisory and speaking and lecture fees. Bjorn Gudbjornsson reports a relationship with nordic pharma that includes: speaking and lecture fees. Espen A. Haavardsholm reports a relationship with Pfizer that includes: speaking and lecture fees. Espen A. Haavardsholm reports a relationship with ucb that includes: speaking and lecture fees. Espen A. Haavardsholm reports a relationship with abbvie that includes: speaking and lecture fees. Dan Nordstrom reports a relationship with MSD that includes: consulting or advisory and funding grants. Dan Nordstrom reports a relationship with BMS that includes: consulting or advisory. Dan Nordstrom reports a relationship with Lilly that includes: consulting or advisory. Dan Nordstrom reports a relationship with Novartis that includes: consulting or advisory. Dan Nordstrom reports a relationship with Pfizer that includes: consulting or advisory. Dan Nordstrom reports a relationship with ucb that includes: consulting or advisory. Merete Lund Hetland reports a relationship with abbvie that includes: funding grants. Merete Lund Hetland reports a relationship with alfasigma that includes: funding grants. Merete Lund Hetland reports a relationship with BMS that includes: funding grants. Merete Lund Hetland reports a relationship with Eli Lilly that includes: funding grants. Merete Lund Hetland reports a relationship with msd that includes: funding grants. Merete Lund Hetland reports a relationship with Pfizer that includes: funding grants and speaking and lecture fees. Merete Lund Hetland reports a relationship with Sandoz Inc that includes: funding grants and speaking and lecture fees. Merete Lund Hetland reports a relationship with Novartis that includes: funding grants and speaking and lecture fees. Merete Lund Hetland reports a relationship with Nordforsk that includes: funding grants. Merete Lund Hetland reports a relationship with UCB that includes: funding grants and speaking and lecture fees. Merete Lund Hetland reports a relationship with MEDAC that includes: speaking and lecture fees. Mikkel Ostergaard reports a relationship with AbbVie Inc that includes: board membership, consulting or advisory, and funding grants. Mikkel Ostergaard reports a relationship with Amgen Inc that includes: funding grants. Mikkel Ostergaard reports a relationship with BMS that includes: board membership, consulting or advisory, and funding grants. Mikkel Ostergaard reports a relationship with Merck & Co Inc that includes: board membership, consulting or advisory, and funding grants. Mikkel Ostergaard reports a relationship with Celgene SL that includes: board membership, consulting or advisory, and funding grants. Mikkel Ostergaard reports a relationship with Eli Lilly and Company that includes: board membership, consulting or advisory, and funding grants. Mikkel Ostergaard reports a relationship with Novartis that includes: board membership, consulting or advisory, and funding grants. Mikkel Ostergaard reports a relationship with ucb that includes: board membership, funding grants, and speaking and lecture fees. Mikkel Ostergaard reports a relationship with Galapagos that includes: board membership and consulting or advisory. Mikkel Ostergaard reports a relationship with Gilead Sciences Inc that includes: board membership and consulting or advisory. Mikkel Ostergaard reports a relationship with Janssen Pharmaceuticals Inc that includes: board membership and consulting or advisory. Mikkel Ostergaard reports a relationship with MEDAC SAS that includes: consulting or advisory. Till Uhlig reports a relationship with galapagos, Pfizer, UCB that includes: speaking and lecture fees. Sokka-Isler Tuulikki reports a relationship with Amgen, Nordic Medicine that includes: funding grants. Sokka-Isler Tuulikki reports a relationship with AbbVie Inc, Lipum, Pfizer, Nordic Medicine, UCB that includes: speaking and lecture fees. Cristina Maglio reports a relationship with Dagens Medicine that includes: speaking and lecture fees. If there are other authors, they declare that they have no known competing financial interests or personal relationships that could have appeared to influence the work reported in this paper.

## Data Availability

Data will be made available on request.
