# Supplementary material for: Acylcarnitine enrichment as a characteristic of rheumatoid arthritis fibroblast-like synoviocyte metabolic fingerprint
Source: J Transl Autoimmun. 2025 Aug 19;11:100310. doi: 10.1016/j.jtauto.2025.100310 (PMC12398893; doi:10.1016/j.jtauto.2025.100310)
Supplement: Multimedia component 1 [file mmc1.docx]

**SUPPLEMENTARY MATERIAL**

**SUPPLEMENTARY METHODS**

*Cell cultures*

Tissue pieces of approximately 1 mm^3^ were minced, treated with 1 mg/ml collagenase/dispase (Roche, Mannheim, Germany) for 1 h at 37°C, and then passed through a cell strainer. The collected cells were washed twice in phosphate-buffered saline (PBS), re-suspended in growth medium containing DMEM medium (high glucose, GlutaMAX™ Supplement from Gibco™, Cat# 10566016) supplemented with 10% heat-inactivated Gibco™ Fetal Bovine Serum, 50 mg/ml Gibco™ Gentamicin, and 100 U/ml Gibco™ Penicillin-Streptomycin at 37°C with 5% CO_2_. All cell culture reagents were purchased from Thermo Fisher Scientific (Waltham, MA, USA), unless otherwise specified.

*Metabolomics analysis by LC-QTOF-MS*

A total of 100 µL of 90% methanol including internal standards (13C9-Phenylalanine, D4-Cholic acid, 13C9-Caffeic Acid, and salicylic acid-D6) were added to each sample. The samples were shaken and centrifuged at +4 °C, 14 000 rpm, for 10 minutes, and 200 µl supernatant was transferred to micro vials for the LC-QTOF-MS analysis. A small aliquot of the remaining supernatants was pooled and used to create quality control samples. MS analysis was run on the quality control samples for identification purposes. Each batch of samples was first analysed in positive mode. After all samples within a batch had been analysed, the instrument was switched to negative mode, and a second injection of each sample was performed. The chromatographic separation was performed on an Agilent 1290 Infinity UHPLC system (Agilent Technologies, Waldbronn, Germany). The compounds were detected with an Agilent 6546 Q-TOF mass spectrometer equipped with a jet stream electrospray ion source operating in positive or negative ion mode. The processing was performed in a targeted fashion. A pre-defined list of metabolites was searched for using the Batch Targeted feature extraction in Masshunter Profinder. An in-house LC-QTOF-MS library built up by authentic standards run on the same system with the same chromatographic and mass-spec settings was used for the processing.

*Western blot*

Whole cell lysates were collected from NI (n=7) and RA (n=9) FLS using RIPA buffer (Cell Signalling, Danvers, MA, USA, Cat# 9806S) supplemented with 1 mM phenylmethylsulfonyl fluoride (Thermo Fisher Scientific, Waltham, MA, USA, Cat# 36978), Protease and Phosphatase Inhibitor Cocktail (Thermo Fisher Scientific, Waltham, MA, USA, Cat# 78443). Protein gel electrophoresis was performed using 10% Mini-PROTEAN TGX Precast Protein Gels (Bio-Rad, Hercules, CA, USA, Cat# 4561034). Then the proteins were transferred to PVDF membranes (Immobilon-FL from Merck KGaA, Darmstadt, Germany, Cat# NWKITFL). Expressions of carnitine palmitoyltransferase 1A (CPT1A), carnitine palmitoyltransferase II (CPT2), CD36, solute carrier family 22 member 5 (OCTN2), and Acyl-CoA Synthetase Long Chain Family Member 5 (ACSL5) were detected using specific primary antibodies and secondary antibodies. The PVDF membranes were blocked in 5% non-fat dry milk/tris-buffered saline (TBS) at room temperature for 1 hour and then incubated in primary antibody dilutions at 4°C overnight. The dilutions of the primary antibodies are carnitine CPT1A (Proteintech, Manchester, UK, Cat# 15184-1-AP) 1:500, CPT2 (Santa Cruz Biotechnology, Cat# sc-377294) 1:100, CD36 (Thermo Fisher Scientific, Waltham, MA, USA, Cat# PA1-16813) 1:500, OCTN2 (Abcam, Cambridge, United Kingdom, Cat# ab180757) 1:1000, ACSL5 (Proteintech, Manchester, UK, Cat# 15708-1-AP) 1:1000 in 1% bovine serum albumin/TBS. Vinculin (Cell Signalling, Danvers, MA, USA, Cat# 13901S) 1:5000 diluted was used as a loading control. Goat anti-rabbit IgG and goat anti-mouse IgG secondary antibodies, HRP conjugated (Thermo Fisher Scientific, Waltham, MA, USA, Cat# 65-6120 and 62-6520) diluted at 1:1000 to 1:10000 in 5% non-fat dry milk/TBS were used to detect the specific bindings.

*Seahorse assays*

**Mitochondrial respiration (Mito Stress test):** One hour before the assay, cells were incubated with XF basal medium supplemented with 10 mM glucose and 4 mM glutamine (pH 7.4) and incubated at 37°C in a CO2-free incubator. The hydrated wells of the sensor cartridge were loaded with oligomycin (final concentration 2 μM, port A) (O4876, Sigma-Aldrich), carbonyl cyanide 4-(trifluoromethoxy) phenylhydrazone (FCCP, C2920, Sigma-Aldrich) (5 μM, port B), and antimycin A (A8674, Sigma-Aldrich) + rotenone (R8875, Sigma-Aldrich) (2 μM port C). OCR was measured during sequential incubations to determine (1) basal respiration, (2) maximal respiration (FCCP), and (3) nonmitochondrial respiration (antimycin A/rotenone). Parameters were calculated following the instructions of Seahorse XF Cell Mito Stress Test Report Generator.

**Fatty acid oxidation (FAO):** After glucose deprivation overnight, the medium was replaced with XF basal medium supplemented with 1 mM glucose and 0.5 mM carnitine (pH 7.4) (for CPT1A function) and incubated for 1 h at 37°C in a CO2-free incubator. The hydrated wells of the sensor cartridge were then loaded with oligomycin, FCCP, and antimycin A + rotenone as described above. Fifteen minutes before measurements, cells were treated with 100 μM etomoxir, a CPT-1 inhibitor (E1905, Sigma-Aldrich). At time 0, a saturating amount of palmitate-BSA (150 µM, XF palmitate–BSA FAO substrate, 102720-100, Agilent Technology) was added. This test reveals the portion of the OCR signal generated by endogenous and total FAO (from endogenous and exogenous FA). These parameters were calculated with normalised ECAR values using the equations given below:

- Basal respiration due to utilisation of exogenous FAs (Last OCR measurement before addition of oligomycin) = Palmitate/ Vehicle – Palmitate/Etomoxir

- Maximal respiration due to utilisation of exogenous FAs (Maximum OCR measurement after addition of FCCP) = Palmitate/ Vehicle – Palmitate/Etomoxir

- Basal respiration due to utilisation of endogenous FAs (Last OCR measurement before addition of oligomycin) = BSA/ Vehicle – BSA/Etomoxir

- Maximal respiration due to utilisation of endogenous FAs (Maximum OCR measurement after addition of FCCP) = BSA/Vehicle – BSA/Etomoxir

**ATP rate assay:** This assay simultaneously measures the rates of ATP production from glycolysis (GlycoATP) and mitochondrial respiration (MitoATP) in live cells. One hour before the assay, cells were incubated with XF basal medium supplemented with 10 mM glucose and 4 mM glutamine (pH 7.4) to measure ATP produced from carbohydrates and with 1 mM glucose/0.5 mM carnitine (pH 7.4) to measure ATP produced from fatty acids (150 µM palmitate added just before running plate). OCRs and ECARs were measured after the sequential addition of oligomycin and antimycin A with rotenone (all at 2 µM). Parameters were calculated following the instructions of Seahorse XF ATP Rate Assay Report Generator.

**Acylcarnitine stimulation experiment:** Exogenous acetylcarnitine, decanoylcarnitine, and stearoylcarnitine were purchased from Cayman Chemical (Ann Arbor, MI, USA, Cat# 16948, 26549, and 26556). NI and RA FLS were seeded in 96-well plates and were allowed to adhere for 24 hours. Then the cells were stimulated with different doses of exogenous acetylcarnitine, decanoylcarnitine, and stearoylcarnitine. At 24 hours after stimulation, cell culture supernatants were collected and IL-6 concentrations were measured using Human IL-6 DuoSet ELISA (Bio-Techne, Minneapolis, MN, USA, Cat# DY206). Cell Counting Kit 8 was purchased from Abcam (Cambridge, UK, Cat# ab228554) and the assay was performed following the manufacturer's instruction. The absorbance was measured at 450 nm using a Spark Cyto plate reader (Tecan Trading AG, Männedorf, Switzerland).

**Supplementary Table 1. Characteristics of non-inflamed controls and patients with RA who donated synovial tissue for FLS isolation.**

| Characteristics | Non-inflamed  (n = 7) | Rheumatoid arthritis  (n = 10) | *p*-value |
| --- | --- | --- | --- |
| Women, n (%) | 3 (43) | 8 (80) | 0.11 |
| Age, years | 41 ± 21 | 65 ± 15 | 0.03 |
| Treatment at the time of surgery |  |  |  |
| Methotrexate |  | 3 (30) |  |
| Methotrexate + infliximab |  | 2 (20) |  |
| Methotrexate + etanercept |  | 1 (10) |  |
| No DMARDs |  | 3 (30) |  |
| Unknown |  | 1 (10) |  |

Continuous variables are expressed as mean ± standard deviation, while categorical variables are expressed as numbers and percentages. *p*-values are provided for a comparison between non-inflamed and rheumatoid arthritis groups by Mann-Whitney test.

DMARDs: disease-modifying antirheumatic drugs.

**Supplementary Table 2: Acylcarnitines enriched in RA FLS compared to NI FLS**

| **Acylcarnitine** | **Chain length** | **Carbon** | **Fold change** | **p** |
| --- | --- | --- | --- | --- |
| 3-hydroxyoctadecenoylcarnitine | Long | C18 | 4.6 | <0.001 |
| Hexanoylcarnitine | Medium | C6 | 3.3 | 0.003 |
| Cervonylcarnitine | Very long | C22  C1 | 3.3 | 0.001 |
| Decanoylcarnitine | Medium | C10 | 3.2 | <0.001 |
| (7Z,10Z,13Z,16Z)-Docosatetraenoylcarnitine | Very long | C22 | 2.9 | 0.003 |
| L-Octanoylcarnitine | Medium | C8 | 2.6 | <0.001 |
| Arachidonoylcarnitine | Long | C20 | 2.4 | 0.001 |
| Docosapentaenoylcarnitine | Very long | C22 | 2.3 | 0.003 |
| L-Acetylcarnitine | Short | C2 | 2.1 | 0.04 |
| Dodecanoylcarnitine | Medium | C12 | 2.0 | 0.01 |
| 2-Hydroxymyristoylcarnitine | Long | C14 | 2.0 | 0.001 |

Acylcarnitine classification is according to carbon chain length. Fold change increase in RA FLS vs NI FLS. p values are from Mann-Whitney test.

*Abbreviations*: *RA,* rheumatoid arthritis; *FLS,* fibroblast-like synoviocytes; *NI,* non-inflamed

**Supplementary Table 3. Characteristics of people with early RA included in the current report, overall and after stratification according to response to treatment at the 24-week follow-up**

| Characteristics | All (n = 220) | | Arm 1 (n = 58) | | Arm 2 (n = 52) | | Arm 3 (n = 56) | | Arm 4 (n = 54) | | *p*-value |  |
| --- | --- | --- | --- | --- | --- | --- | --- | --- | --- | --- | --- | --- |
| Women, n (%) | 153 (70) | | 40 (69) | | 33 (63) | | 41 (73) | | 39 (72) | |  |  |
| Age, years | 54 ± 15 | | 56 ± 14 | | 54 ± 17 | | 55 ± 17 | | 53 ± 12 | |  |  |
| BMI | 26 ± 5 | | 26 ± 5 | | 25 ± 4 | | 26 ± 5 | | 27 ± 5 | |  |  |
| Current smokers, n (%) | 45 (20) | | 11 (19) | | 13 (25) | | 9 (16) | | 12 (22) | |  |  |
| RF positive, n (%) | 162 (74) | | 42 (72) | | 38 (73) | | 41 (73) | | 41 (76) | |  |  |
| ACPA positive, n (%) | 181 (82) | | 48 (83) | | 42 (81) | | 44 (79) | | 47 (87) | |  |  |
| Symtom duration, days | 212 ± 166 | | 229 ± 181 | | 184± 153 | | 202± 142 | | 232± 182 | |  |  |
| Time since diagnosis, days | 8 ± 26 | | 6 ± 10 | | 8 ± 24 | | 8 ± 11 | | 12 ± 44 | |  |  |
| Remission, n (%) | 98 (45) | | 25 (43) | | 22 (42) | | 26 (46) | | 25 (46) | |  |  |
|  | Baseline | 24-week | Baseline | 24-week | Baseline | 24-week | Baseline | 24-week | Baseline | 24-week |  | |
| CDAI | 29 ± 12 | 5.6 ± 5.6 | 29 ± 13 | 5.4 ± 5.5 | 29 ± 13 | 5.1 ± 4.9 | 31 ± 11 | 5.6 ± 5.8 | 28 ± 11 | 6.2 ± 6.3 | <0.0001 | |
| ESR, mm/h | 34 ± 25 | 11 ± 11 | 37 ± 26 | 13 ± 11 | 38 ± 27 | 13 ± 10 | 36 ± 26 | 15 ± 13 | 26 ± 16 | 4.0 ± 6.2 | <0.0001 | |
| CRP, mg/L | 22 ± 31 | 2.5 ± 4.2 | 28 ± 40 | 4.0 ± 5.7 | 27 ± 39 | 1.7 ± 1.8 | 22 ± 23 | 3.0 ± 5.0 | 12 ± 13 | 1.1 ± 2.1 | <0.0001 | |
| DAS28-ESR | 5.5 ± 1.1 | 2.3 ± 1.2 | 5.6 ± 1.2 | 2.4 ± 1.1 | 5.5 ± 1.3 | 2.5 ± 1.0 | 5.7 ± 1.0 | 2.6 ± 1.1 | 5.4 ± 1.1 | 1.7 ± 1.2 | <0.0001 | |
| DAS28-CRP | 5.1 ± 1.1 | 2.2 ± 1.0 | 5.1 ± 1.2 | 2.3 ± 1.0 | 5.1 ± 1.2 | 2.1 ± 0.8 | 5.2 ± 1.0 | 2.3 ± 1.0 | 5.0 ± 1.0 | 2.2 ± 1.0 | <0.0001 | |

Continuous variables are expressed as mean ± standard deviation, while categorical variables are expressed as number and percentages. *p*-values are provided for a comparison before and after treatment in all participants as well as in each treatment arm by paired t-test.

BMI: body mass index, RF: rheumatoid factor, ACPA: anti-citrullinated peptide antibody, CDAI: Clinical Disease Activity Index, ESR: Erythrocyte sedimentation rate, CRP: C-reactive protein, DAS28-ESR: Disease activity score of 28 joints (ESR-based), DAS28-CRP: Disease activity score using 28 joint counts (CRP-based)

**SUPPLEMENTARY FIGURES**

**
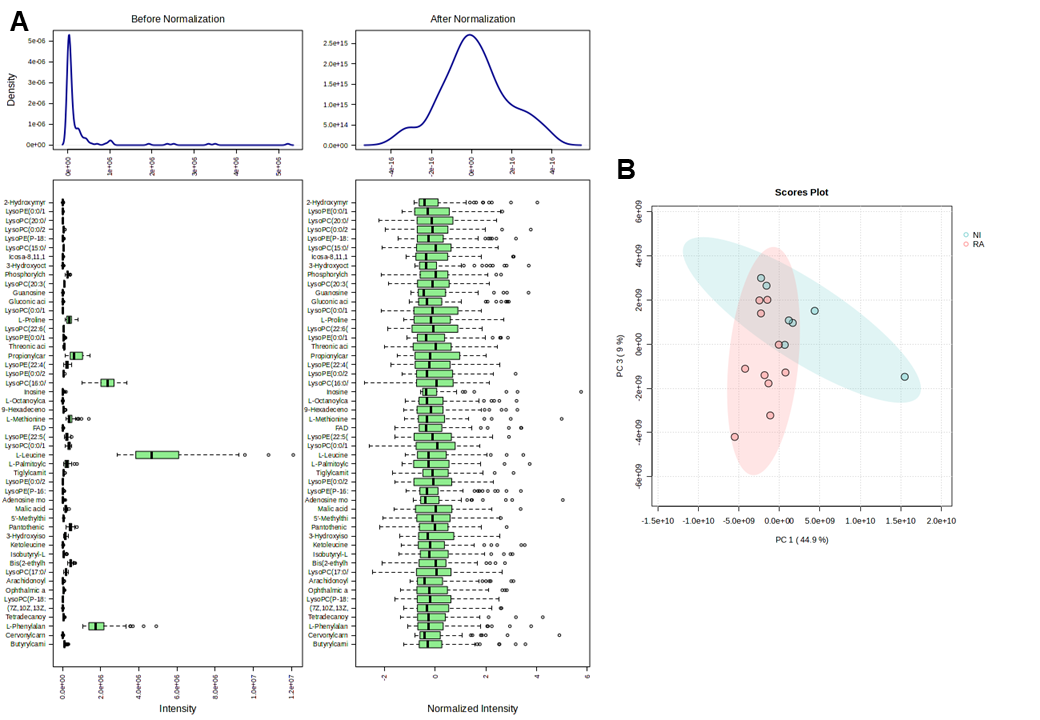
**

**Supplementary Figure 1**: **FLS metabolomics analysis**. **A**. Results of metabolite peak intensity normalisation with auto scaling (mean-centred and divided by the standard deviation of each variable). **B.** Principal component analysis (PCA) of principal component 1 and principal component 3, showing each individual sample and the 95% confidence interval.


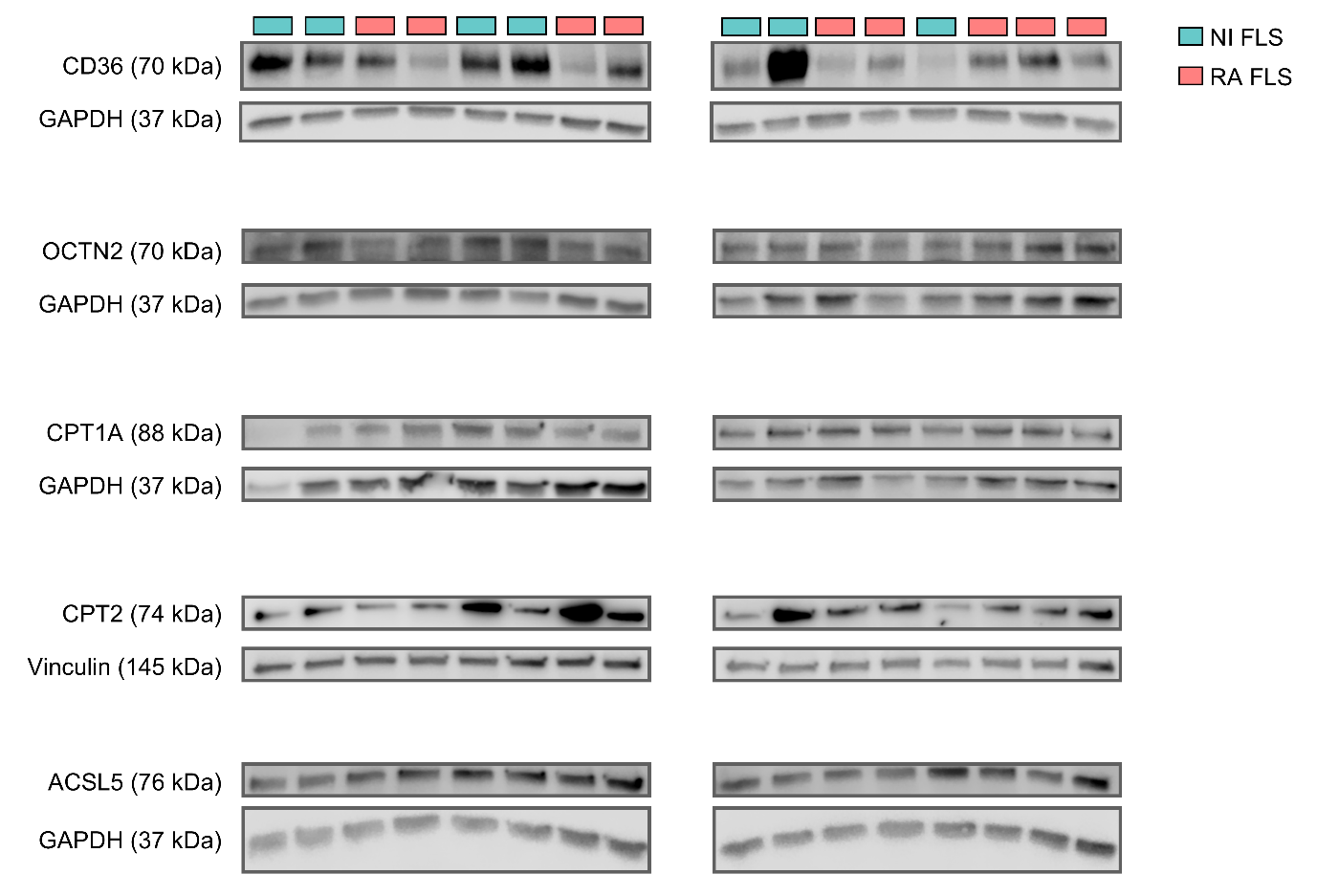


**Supplementary Figure 2: Original pictures of western blot.**

**
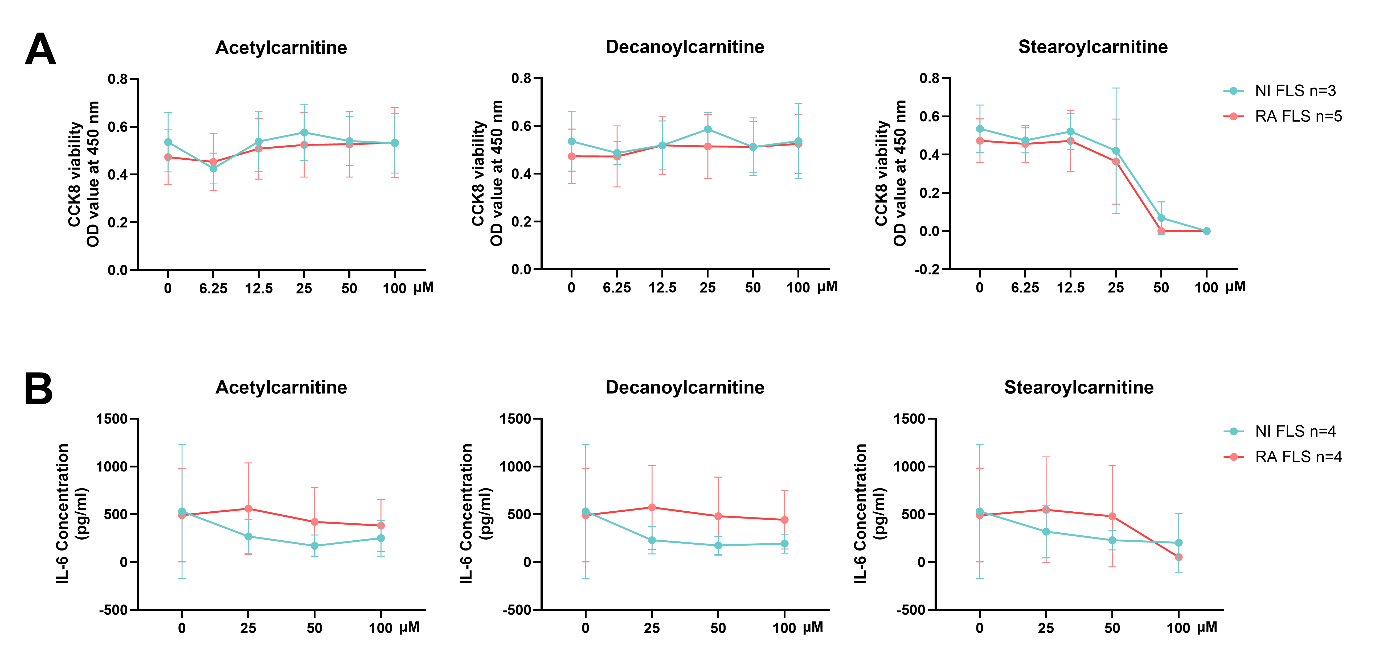
**

**Supplementary Figure 3: Cell viability and interleukin-6 (IL-6) production in NI-FLS stimulated by acylcarnitines.** NI FLS were stimulated with different doses of three exogenous acylcarnitines for 24 hours and the cell viability was measured using Cell Counting Kit 8 assay (A) and the concentrations of IL-6 in cell culture supernatants were measured using ELISA (B).

**
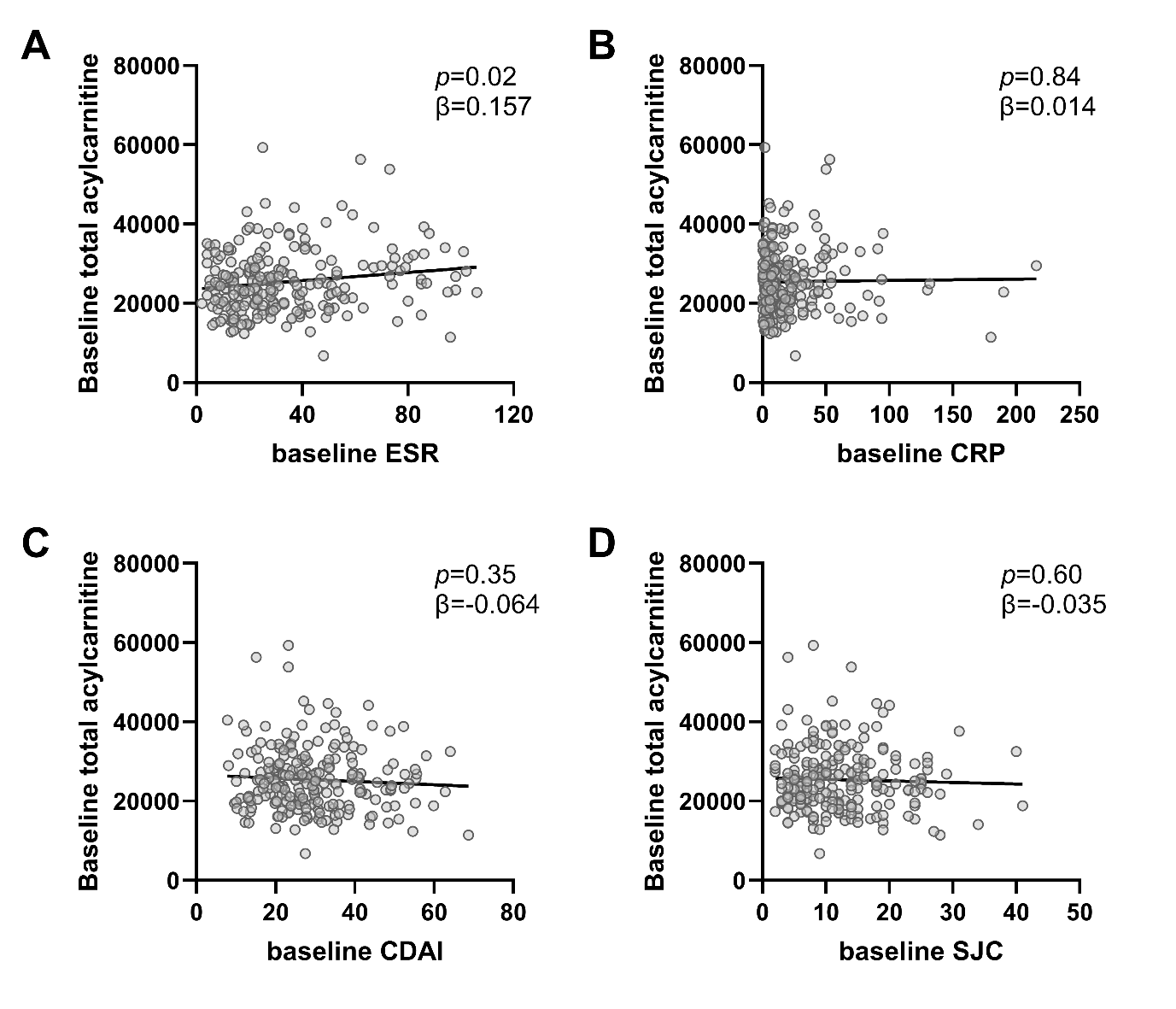
**

**Supplementary Figure 4: Linear regression showing associations between baseline total acylcarnitine and baseline A. ESR, B. CRP, C. CDAI, or D. SJC.**

**
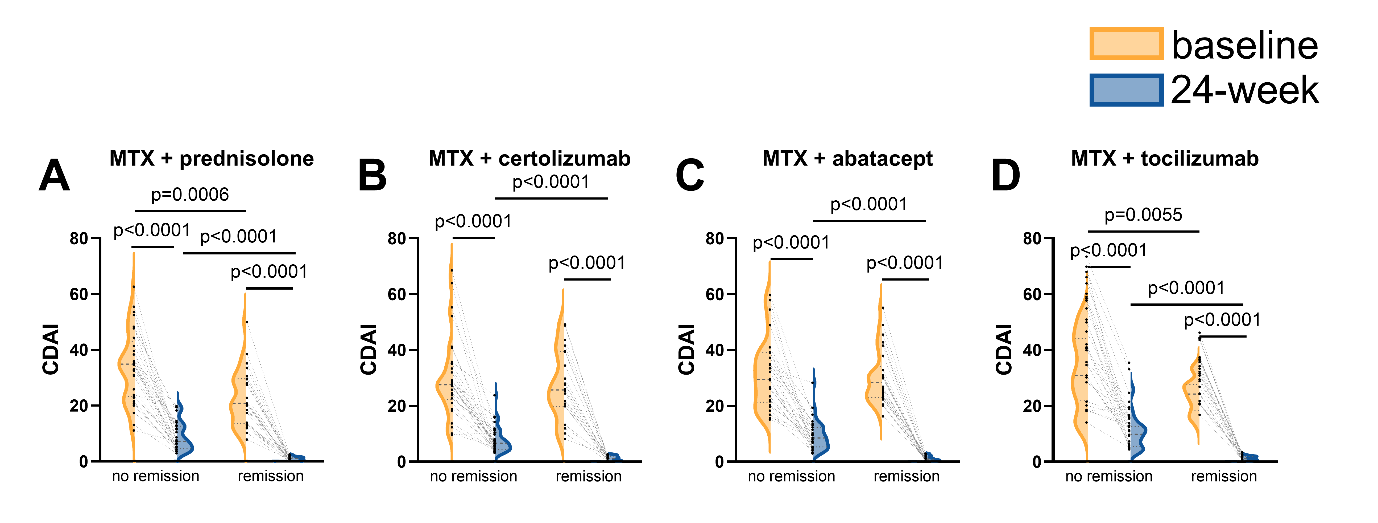
**

**Supplementary Figure 5: CDAI change in each treatment arm at 24-week follow-up.** Participants treated with **A.** MTX + prednisolone, **B.** MTX + certolizumab-pegol, **C.** MTX + abatacept, and **D.** MTX + tocilizumab were divided into remission (CDAI≤2.8 at week 24) and no remission groups. Significance was determined using paired t-test for comparisons between baseline and 24-week and unpaired t-test for comparisons between no remission and remission.
